# Changes in US Hospital Financial Performance During the COVID-19 Public Health Emergency

**DOI:** 10.1001/jamahealthforum.2023.1928

**Published:** 2023-07-14

**Authors:** Risha Gidwani, Cheryl L. Damberg

**Affiliations:** 1 RAND Corporation, Santa Monica, California; 2 Department of Health Policy and Management, UCLA Fielding School of Public Health, Los Angeles, California

## Abstract

**Importance:**

The COVID-19 public health emergency (PHE) caused substantial changes in hospital operations. The net effect of these changes on hospital financial performance is unclear.

**Objective:**

To evaluate changes in hospital financial performance before and during the COVID-19 PHE.

**Design, Setting, and Participants:**

This longitudinal within-hospital cohort analysis from 2017 to 2021 used national RAND hospital data merged with American Community Survey data. A total of 4223 short-term acute care or critical access hospitals in the US with financial data spanning 2017 to 2021 were evaluated.

**Exposure:**

Financial performance during the first 2 years of the PHE.

**Main Outcomes and Measures:**

The main outcome was PHE financial distress calculated based on net operating income (operating revenue minus operating expenses). Within-hospital changes in net operating income over time were evaluated with and without COVID-19 relief funding. From henceforth, 2020/2021 means the weighted average financial performance for both calendar year 2020 and 2021. Hospitals were characterized as having new financial distress if (1) their average 2020/2021 net operating income was negative and (2) the average 2020/2021 net operating income was less than that hospital’s pre-2020 net operating income. Predictors of new financial distress were evaluated using logistic regression and predictors of COVID-19 relief using 2-part models.

**Results:**

In this sample of 4423 hospitals, 3529 (80.0%) received PHE funds during 2020/2021. A total of 846 (19.1%) were located in a census tract with more than 20% Hispanic residents. Of the total number of hospitals, 720 (16.3%) of hospitals had PHE financial distress, whereas 2047 (46.3%) had PHE financial distress after excluding COVID-19 relief funding from net operating income. The majority of hospitals (n = 3337; 74.8%) had a positive net operating income across 2020/2021, with 785 (17.8%) hospitals moving from a negative pre-2020 to a positive 2020/2021 net operating income. In adjusted analyses, hospitals treating a higher proportion of Hispanic populations were more likely to have PHE distress (adjusted odds ratio, 1.3; 95% CI, 1.1-1.6; *P* = .02). Median (IQR) operating margins from 2020/2021 were at an all-time high of 6.5% (0.2%-13.3%) compared with pre-2020 operating margins of 2.8% (−2.8% to 9.3%).

**Conclusions and Relevance:**

In this cohort study of US hospitals, the large majority had positive financial performance during 2020/2021, partly due to COVID-19 relief funds. However, hospitals serving Hispanic populations had substantially worsened financial performance during 2020/2021, even after accounting for COVID-19 relief. That COVID-19 relief funding aided in operating margins reaching all-time highs indicates funding amounts may have been larger than was necessary for many hospitals. With COVID-19 relief funding ending yet COVID-19 related continuing to affect hospital expenses, ongoing monitoring of hospital financial performance is vital to ensure patients retain access to care.

## Key Points

**Question:**

How did the financial position of hospitals change during the COVID-19 public health emergency?

**Findings:**

In this national cohort study of 4423 hospitals, 3337 (75%) hospitals had a positive net operating income during 2020/2021, and 720 (16%) experienced new financial distress. Hospitals serving Hispanic populations were more likely to experience financial distress, even after receiving public health emergency funding; however, COVID-19 relief funding aided in hospital net operating margins reaching all-time highs.

**Meaning:**

Although the majority of US hospitals were financially healthy across 2020 and 2021, partly due to the provision of COVID-19 relief funds, the size of COVID-19 relief funds may have been larger than was necessary for many hospitals.

## Teaser

This cohort study of US hospitals evaluates the changes in hospital financial performance before and during the COVID-19 public health emergency.

## Introduction

The COVID-19 pandemic imposed numerous changes to hospitals’ operations, changes that likely had differential effects on hospital financial performance. On the one hand, hospitals expended additional resources in activities such as creating COVID-19–specific wards, converting existing rooms to isolation units or negative pressure rooms, increasing purchases of personal protective equipment, increasing the frequency and intensity of cleaning protocols, and hiring contract labor such as nurses or physicians.^1,2^ On the other hand, operating revenues may have decreased because non–COVID-19 services, such as elective surgeries and imaging, were delayed or canceled to create capacity to care for COVID-19 patients during pandemic surges, inducing downward pressure on hospital financial performance. However, hospitals may have received substantial COVID-19 funding relief from the federal government to offset costs, including through the Coronavirus Aid, Relief, and Economic Security Act (CARES Act), specifically, the provisions that established the Provider Relief Fund or the 20% Medicare Severity Diagnosis-Related Group (MS-DRG) add-on payment for the treatment of COVID-19 patients.^3,4^ Furthermore, a hospital’s uncompensated care burden may have decreased because of the Health Resources and Services Administration (HRSA) COVID-19 claims reimbursement fund, which allowed providers (ie, hospitals, health systems, and physicians) treating uninsured COVID-19 patients to receive reimbursement for their care.^5^ Other concurrent changes, such as a robustly performing stock market in the years before and during the first year of the pandemic may have bolstered the finances of hospitals with an investment portfolio.^6^

In the first years of the pandemic, hospitals asserted they were incurring substantial financial losses as a result of responding to the COVID-19 public health emergency (PHE) and expressed deep concerns about their financial stability.^7,8^ Yet limited empirical evidence exists regarding the net effects of these concurrent changes on the financial performance of hospitals. Understanding how COVID-19 influenced the financial health of hospitals is an important policy question, as ensuring patient access to care and the ability to deliver high-quality care requires financially-healthy hospitals. From henceforth, 2020/2021 means the weighted average financial performance for both calendar year 2020 and 2021. Here, we evaluate the 2020/2021 financial performance of the population of US hospitals relative to their own prepandemic performance to assess how COVID-19 was associated with hospital financial health, including how PHE funding was associated with hospital financial health.

## Methods

In this cohort study of US hospitals, we obtained financial data from the RAND Hospital Data for the calendar years 2017 to 2021.^9^ Hospitals contracting with the Medicare program are required to submit Healthcare Cost Report Information System (HCRIS) data annually to the Centers for Medicare & Medicaid Services (CMS). The RAND Corporation cleans and maintains the HCRIS data, producing a data set called the RAND Hospital Data, which was used for the present analysis. Hospitals can submit financial data according to their own fiscal year, which may correspond to the calendar year. When submitted data crosses calendar years, RAND prorates the data to the appropriate calendar year. RAND Hospital Data encompass all US hospitals except for Veterans Health Administration hospitals, Indian Health Services hospitals, and some children’s hospitals.

The present hospital cohort consisted of short-term acute care hospitals (subject to Medicare inpatient prospective payment system [IPPS] payments ) and critical access hospitals; there are 4628 such hospitals in the data. For study inclusion, hospitals had to report at least 2 years of pre-PHE data (2017 to 2019). We excluded hospital years with negative, missing, or $0 values reported for operating revenue or operating expenses. As of April 1, 2023, 98.3% of hospitals reported full 2020 data, and 71.1% of hospitals reported full 2021 data. The final sample contained 4423 hospitals.

The RAND Human Subjects Protection Committee determined the study to be exempt from review. This study follows the Strengthening the Reporting of Observational Studies in Epidemiology (STROBE) reporting guideline for cohort studies.^10^

### Outcomes

#### New PHE Distress

The primary goal of the present study was to identify hospitals in new financial distress during the COVID-19 PHE, based on their net operating income. *Net operating income* is calculated as operating revenue minus operating expenses. It indicates the hospital’s financial performance due to patient-care services and governmental disproportionate share hospital (DSH) funds. In colloquial terms, net operating income can be considered the organization’s profit before taxes and other deductions are applied. For 2020/2021, operating revenue, and therefore net operating income, includes COVID-19 relief funding, such as money from the Provider Relief Fund. We ran separate analyses excluding COVID-19 funding from net operating revenue, focusing only on net income from patient services (which includes DSH payments), to gauge the extent to which COVID-19 funding alleviated hospitals’ financial distress.

We considered a hospital to be in new PHE distress if it had both (1) a weighted average net operating income across 2020/2021 that was negative and (2) the weighted average 2020/2021 net operating income was less than the hospital’s pre-2020 weighted average net operating income. Pre-2020 net operating income was calculated using a weighted average of net operating income from 2017 to 2019. Weighted averages are used by the Medicare Payment Advisory Commission (MedPAC) in their reporting to Congress.^11^ Thus, a hospital was in new PHE distress if it had both worse financial performance in 2020/2021 than in previous years, and that worse financial performance resulted in a 2020/2021 negative net operating income. Those with negative financial performance pre-2020 that subsequently improved and those with positive financial performance pre-2020 that declined but did not subsequently move negative were not considered to be in new PHE distress.

#### Operating Margin

To facilitate comparisons across hospitals, whose incomes can vary substantially in size, we also evaluated operating margins. *Operating margin* is defined as the difference between operating revenues and operating expenses (ie, net operating income) divided by operating revenues. A hospital with a net operating income of $4 million earned on $100 million of revenue would have an operating margin of 4%, as would a hospital with a net operating income of $32 million earned on $800 million of revenue. We evaluated a weighted average of 2020/2021 margins as well as within-hospital changes in operating margins from pre-2020 to 2020/2021, using a weighted average of all available data from 2017 to 2019 to define pre-2020 operating margins.

### Statistical Analysis

We evaluated hospital characteristics associated with new PHE distress using logistic regression models, with 1 set of models that included COVID-19 relief funding and another set that excluded such funding. Covariates included hospital type (acute care or critical access), teaching status, DSH status (categorized as No DSH, Medicaid DSH, Medicare DSH, or both), belonging to a health system, uncompensated care as a proportion of operating expenses (categorized as 0%, >0% to <5%, 5% to <10%, 10% to <15%, or ≥15%), for-profit status (categorized as not-for-profit, for-profit, and government), and rurality (categorized as rural, urban, and highly urban). Rural was defined as all non-metropolitan areas, urban was defined as counties in metropolitan areas, and highly urban was defined as counties in metropolitan areas with 1 million or more residents by collapsing 9-level rural-urban continuum codes. Additionally, we merged data on race, ethnicity, and poverty from the American Community Survey from 2015 to 2019 and incorporated as model covariates 3 indicator variables characterizing whether the hospital was located in a census tract where more than 20% of the population was Black; Hispanic; or lived below the poverty line. Black and Hispanic groups were evaluated because they often experience obstacles in accessing high-quality health care, including structural racism, and, in general, experience a distinct array of social determinants of health that can impede access to health care. In the model where COVID-19 funding was included in net operating income, we also included a covariate capturing COVID-19 funding as a proportion of operating expenses, characterized as a categorical variable with levels of 0%, >0% to 2%, 2% to <5% and ≥5%. We generated predicted probabilities of PHE financial distress using the margins postestimation command.

Lastly, we evaluated predictors of receiving COVID-19 relief funding using 2-part models, using the twopm command in Stata, specifying a logistic model for the first model and a generalized linear model with a log link and a gamma family for the second part and leveraging the margins commands to generate combined estimates from the 2 models.^12^

All analyses were conducted in Stata, version 16 (StataCorp LLC). All statistical tests were 2-sided and used an α of .05.

## Results

Of the 4423 hospitals in the sample, the majority (n = 3094; 70.1%) were short-term acute care hospitals (Table 1). Most hospitals had DSH status, although only half (n = 2238; 50.6%) received such a designation and its accompanying payments for both their Medicare and Medicaid patients. Approximately one-quarter of hospitals were teaching hospitals (n = 1223; 27.7%), and almost three-quarters (n = 3123; 70.6%) belonged to a health care system. Rural hospitals made up the greatest proportion of the cohort (n = 1686; 38.1%), followed by highly urban (n = 1426; 32.2%) and urban (n = 1311; 29.6%) hospitals. Most (n = 3529; 80.0%) hospitals received PHE funding in 2020 and/or 2021, where PHE funds represented a median (IQR) of 3.1% (1.3%-6.0%) of their 2020/2021 operating expenses. With respect to ownership, 2631 (59.5%) were not-for-profit, 823 (18.6%) were for-profit, and 969 (21.9%) were government owned.

**Table 1.** Hospital Characteristics^a^

Characteristic (n = 4423)	No. (%)
Hospital type	
Short-term acute care	3094 (70.1)
Critical access	1323 (30.1)
Disproportionate share hospital status	
None	812 (18.4)
Medicare only	411 (9.3)
Medicaid only	962 (21.8)
Medicare and Medicaid	2238 (50.6)
Rurality	
Rural	1686 (38.1)
Urban	1311 (29.6)
Highly Urban	1426 (32.2)
Teaching hospital	
Yes	1223 (27.7)
Hospital belongs to a health system	
Yes	3123 (70.6)
Characteristics of census tract of hospital	
>20% Black residents	684 (15.5)
>20% Hispanic residents	846 (19.1)
>20% of residents below poverty line	660 (14.9)
Received PHE funds in 2020 or 2021	
Yes	3529 (80.0)
Median PHE funds as a proportion of 2020/2021 operating expenses (IQR)^b^	3.1 (1.3 to 6.0)
Negative operating margin, 2017-2019	1600 (36.3)
Negative operating margin, 2020-2021	1052 (23.8)
Median (IQR) operating margin, 2017-2019, %	2.8 (−2.8 to 9.3)
Median (IQR) operating margin, 2020-2021, %	6.5 (0.2 to 13.3)
Median (IQR) operating margin exclusive of PHE funding, 2020-2021, %	−1.0 (−3.5 to 1.8)
Median (IQR) percent change in operating margin from pre-2020 to 2020/2021, %^c^	−5.9 (−98.1 to 63.6)

^a^ Data from 2019 unless otherwise specified.

^b^ Non-zero dollar values only.

^c^ Percent change is distinct from percentage point change.

### Financial Performance

Before 2020, the weighted median (IQR) operating margin of a US short-term acute care or critical access hospital was 2.8% (−2.8% to 9.3%). During 2020/2021, overall, the operating margin improved to a weighted median (IQR) and all-time high of 6.5% (0.2%-13.3%). Excluding COVID-19 relief funding from operating margin, the 2020/2021 weighted median (IQR) operating margin was −1.0% (−3.5% to 1.8%).

Overall, 3337 hospitals, or 74.8% of the sample, had a positive net operating income in 2020/2021 (Table 2). Of the 25.2% of hospitals that had a negative 2020/2021 weighted net operating income, the majority (74.6%; n = 832) had a negative net operating income before 2020. When excluding COVID-19 relief funding from net operating income, 2608 (36.3%) had a positive net operating income in 2020/2021 (Table 2). In this sample, 720 (16.28%) of hospitals had PHE financial distress, whereas 2047 (46.28%) had PHE financial distress after excluding COVID-19 relief funding from net operating income.

**Table 2.** Change in Hospital Financial Performance^a^

Pre-2020	2020-2021	Hospitals, No. (%)	Median (IQR)		
			Net operating income, 2017-2019, millions of $	Net operating income, 2020-2021, millions of $	Within-hospital change in net operating income, millions of $
**Based on net operating income (including COVID-19 relief funds)**					
Negative	Negative	832 (18.8)	−3.6 (−13.1 to −=1.6)	−4.0 (−13.2 to −1.3)	−0.1 (−3.3 to 1.9)
Negative	Positive	785 (17.8)	−1.2 (−3.3 to −0.5)	2.3 (1.0 to 5.0)	4.1 (2.1 to 9.1)
Positive	Positive	2522 (57.0)	9.9 (2.4 to 30.4)	13.1 (4.3 to 38.4)	2.6 (−0.4 to 9.4)
Positive	Negative	284 (6.4)	3.7 (1.0 to 12.6)	−4.2 (−11.5 to −1.2)	−8.9 (−26.1 to −3.4)
Total	NA	4.423 (100)	1.6 (−1.1. to 15.1)	3.9 (−0.03 to 19.2)	1.9 (−1.1 to 7.3)
**Based on net patient services income (excluding COVID-19 relief funds)**					
Negative	Negative	2362 (53.4)	−4.6 (−14.2 to −2.0)	−5.8 (−20.5 to −2.4)	−1.0 (−5.7 to 0.4)
Negative	Positive	260 (5.9)	−2.5 (−7.4 to −0.8)	3.0 (1.0 to 9.6)	6.9 (2.9 to 18.9)
Positive	Positive	1348 (30.5)	13.3 (4.3 to 33.7)	12.9 (4.2 to 36.4)	0.4 (−4.1 to 6.0)
Positive	Negative	453 (10.2)	4.3 (1.0 to 13.8)	−4.8 (−17.2 to −1.4)	−11.9 (−35.3 to −3.8)
Total	NA	4.423 (100)	−1.1 (−5.9 to 6.3)	−2.0 (−9.2 to 4.4)	−0.8 (−6.5 to 1.8)

Abbreviation: NA, not applicable.

^a^ All data are derived from weighted averages.

In this data set, 720 (16.3%) all hospitals experienced PHE financial distress. These 720 hospitals experienced substantial losses in net operating income from pre-2020 to 2020/2021: a median (IQR) within-hospital decline of $5.2 million (−$16.8 million to −$1.3 million). When excluding COVID-19 relief funds from net operating income, 2047 (46.3%) of hospitals would have been in new PHE distress. There were 2376 (53.7%) hospitals that did not experience new PHE distress even without COVID-19 relief funds; however, 1817 (76.5%) of these hospitals received COVID-19 funding.

The median (IQR) within-hospital change in net operating income was $1.9 million (−$1.1 million to $7.0 million) from pre-2020 to 2020/2021 (and −$2.0 million [−$9.2 million to $4.4 million] if excluding COVID-19 funding). Of the 4423 hospitals in the sample, 3012 (68.1%) had an improvement in net operating income from pre-2020 to 2020/2021 (and 1769 [40.0%] if excluding COVID-19 funding). The median (IQR) 2020/2021 net operating income for the 3012 hospitals that improved their financial performance was $5.3 million ($1.4 million-$23.9 million). The median (IQR) change in net operating income from pre-2020 to 2020/2021 for these hospitals was $4.4 million ($1.8 million-$11.9 million). The median (IQR) 2020/2021 net operating income for the 1411 hospitals whose financial performance declined was −$0.1 million (−$6.2 million to $9.4 million). The median (IQR) change in net operating income from pre-2020 to 2020/2021 for these was −$4.6 million (−$1.3 million to −$14.3 million). Importantly, 785 (17.8%) hospitals improved their net operating income from negative in pre-2020 to positive in 2020/2021 (260 [5.9%] hospitals, if excluding COVID-19 funding). A small minority of hospitals (n = 284; 6.4%) fared substantially worse in 2020/2021 compared with their prior performance and had new negative net operating income; these hospitals suffered considerable financial losses with a median within-hospital decline in net operating income of $8.9 million.

In adjusted analyses (Table 3), hospitals located in census tracts in which 20% or more of the population identified as Hispanic were more likely to experience PHE financial distress (adjusted odds ratio [aOR], 1.29; 95% CI, 1.05-1.60; *P* = .02; adjusted probability of PHE financial distress, 19.1% if having a larger proportion of Hispanic residents in the census tract and 15.5% if not). Conversely, health system-affiliated hospitals were significantly less likely to experience new PHE distress compared with hospitals not part of a health system (aOR, 0.79; 95% CI, 0.63-0.99; *P* = .04, adjusted probability of PHE financial distress, 15.4% if system affiliated and 18.7% if unaffiliated). For-profit hospitals were significantly less likely to experience new PHE distress compared with not-for-profit hospitals (aOR, 0.57; 95% CI, 0.44-0.72, *P* < .001, adjusted probability of new PHE distress was 10.8% for for-profit hospitals and 17.5% for not-for-profit hospitals). Hospitals with a greater proportion of COVID-19 funding relative to their operating expenses were also significantly less likely to experience new PHE distress, with a greater proportion of COVID-19 funding being associated with a lower likelihood of distress (Table 4). Model covariates did not exhibit collinearity (see eTable in Supplement 1).

**Table 3.** Regression Models, Likelihood of New Public Health Emergency (PHE) Distress (Including COVID-19 Relief Funds)^a^

	Odds ratio (95% CI)^b^	*P* value	Adjusted probability of new PHE distress, %
Constant	0.33 (0.19-0.57)	NA	NA
Hospital type [reference = short-term acute care]	NA	NA	17.9
Critical access hospital	0.64 (0.45-0.90)	.01	12.3
Rurality [reference = rural]	NA	NA	15.0
Urban	1.04 (0.83-1.31)	.72	15.6
Highly urban	1.26 (0.98-1.61)	.07	18.1
Health System [reference = no]	NA	NA	18.7
In a health system	0.79 (0.63-0.99)	.04	15.4
DSH status [reference = no DSH]	NA	NA	14.2
Medicare-only	1.20 (0.81-1.78)	.37	16.5
Medicaid-only	1.34 (0.99-1.80)	.06	18.0
Medicare and Medicaid	1.18 (0.84-1.65)	.35	16.2
Uncompensated care as a proportion of operating expenses [reference = 0%]	NA	NA	18.7
<5%	0.81 (0.49-1.33)	.40	15.8
5-<10%	0.75 (0.45-1.24)	.26	14.8
10-<15%	0.98 (0.58-1.66)	.95	18.5
≥15%	1.03 (0.60-1.78)	.92	19.2
PHE funding as a proportion of operating expenses [reference = 0%]	NA	NA	22.3
>0%-<2%	0.74 (0.60-0.93)	.01	17.7
2%-<5%	0.56 (0.44-0.71)	<.001	14.1
≥5%	0.47 (0.37-0.61)	<.001	12.1
Black population [reference = <20%]	NA	NA	16.0
≥20% Black population	1.15 (0.90-1.46)	.28	17.8
Population in poverty [reference = <20%]	NA	NA	15.8
≥20% population below the poverty line	1.26 (0.98-1.63)	.07	19.0
Hispanic population [reference = <20%]	NA	NA	15.5
20% Hispanic population	1.30 (1.05-1.60)	.02	19.1
Teaching hospital [reference = no]	NA	NA	15.8
Teaching hospital	1.13 (0.92-1.38)	.25	17.4
Ownership [reference = not-for-profit]	NA	NA	17.5
For-profit	0.57 (0.44-0.72)	<.001	10.8
Government	1.10 (0.88-1.37)	.40	18.8

Abbreviation: DSH, disproportionate share hospital; NA, not applicable.

^a^ PHE distress is defined as having both (1) a negative 2020/2021 net operating income and (2) a 2020/2021 net operating income that was less than that hospital’s pre-2020 net operating income.

^b^ Odds ratios greater than 1 indicate a higher likelihood of experiencing PHE distress.

**Table 4.** Regression Model, Likelihood of New Public Health Emergency (PHE) Distress, Exclusive of COVID-19 Relief Funding^a^

	Odds ratio (95% CI)^b^	*P* value	Adjusted probability of new PHE distress, %
Constant	1.24 (0.82-1.89)	NA	NA
Hospital type [reference = short-term acute care]	NA	NA	44.5
Critical access hospital	1.30 (1.00-1.69)	.05	50.6
Rurality [reference = rural]	NA	NA	46.8
Urban	0.85 (0.72-1.01)	.06	43.2
Highly urban	1.08 (0.90-1.30)	.42	48.6
Health System [reference = no]	NA	NA	59.0
In a health system	0.47 (0.40-0.55)	<.001	41.0
DSH status [reference = no DSH]	NA	NA	40.3
Medicare-only	1.36 (1.00-1.85)	.05	47.3
Medicaid-only	1.00 (0.81-1.22)	.96	40.2
Medicare and Medicaid	1.60 (1.24-2.07)	<.001	51.0
Uncompensated care as a proportion of operating expenses [reference = 0%]	NA	NA	50.8
<5%	0.98 (0.66-1.45)	.92	50.3
5-<10%	0.73 (0.49-1.09)	.12	43.6
10-<15%	0.75 (0.50-1.14)	.18	44.2
≥15%	0.78 (0.51-1.20)	.26	45.0
Black population [reference = <20%]	NA	NA	43.4
≥20% Black population	1.30 (1.07-1.58)	.01	51.4
Population in poverty [reference = <20%]	NA	NA	46.1
≥20% population below the poverty line	1.05 (0.86-1.28)	.65	47.2
Hispanic population [reference = <20%]	NA	NA	46.6
≥20% Hispanic population	0.94 (0.79-1.12)	.50	45.2
Teaching hospital [reference = no]	NA	NA	44.4
Teaching hospital	1.34 (1.14-1.57)	<.001	51.1
Ownership [reference = not-for-profit]	NA	NA	48.1
For-profit	0.46 (0.38-0.55)	<.001	30.6
Government	1.32 (1.12-1.56)	<.001	54.8

Abbreviations: DSH, disproportionate share hospital; NA, not applicable.

^a^ PHE distress is defined as having both (1) a negative 2020/2021 net operating income and (2) a 2020/2021 net operating income that was less than that hospital’s pre-2020 net operating income.

^b^ Odds ratios greater than 1 indicate a higher likelihood of experiencing PHE distress.

The second model excluded COVID-19 funding from net operating income and therefore calculations of new PHE distress (Table 4). Absent COVID-19 funding, health system affiliation remained protective against new PHE distress (aOR, 0.47; 95% CI, 0.40-0.55; *P* < .001), as did being a for-profit hospital (aOR, 0.46; 95% CI, 0.38-0.55, *P* < .001). Four factors emerged as being newly predictive of new PHE distress: government ownership (aOR, 1.32; 95% CI, 1.12-1.56; *P* < .001); teaching hospital status (aOR, 1.34; 95% CI, 1.14-1.57; *P* < .001); location in a census tract where 20% or more of the population was Black (aOR, 1.30; 95% CI, 1.07-1.58; *P* = .01); and having Medicare and Medicaid DSH status (aOR, 1.60; 95% CI, 1.24-2.07; *P* < .001). All other variables retained the same level of significance as in the prior model reported in Table 4. Thus, COVID-19 funding helped government-owned hospitals, teaching hospitals, hospitals serving larger Black populations, and those with Medicare and Medicaid DSH status move from financial distress to an absence of financial distress, as indicated by the difference in regression results between Tables 3 and 4.

Finally, we evaluate predictors of receiving COVID-19 relief funding across 2020/2021 (Table 5). As 80.0% of hospitals received some COVID-19 funding during this time, we present results from a 2-part model, which indicates the amount of funding received conditional on receipt of any funding. Results indicate critical access hospitals received on average, $3.1 million less in COVID-19 relief funding compared with short-term acute care hospitals (95% CI, −$4.4 million to −$1.9 million; *P* < .001). However, regression results indicate that the funding received by critical access hospitals was sufficient to move them from PHE financial distress to no PHE financial distress (Tables 3 and 4). Hospitals with uncompensated care that represented 10% or more of their operating expenses in 2019 were less likely to receive COVID-19 funding compared with those that reported no uncompensated care burden. Health system-affiliated hospitals received an average of $1.2 million less in COVID-19 relief funding compared with hospitals not affiliated with a health system (95% CI, −2.3 million to −0.1 million; *P* = .03). For-profit hospitals received on average $5.4 million less than not-for-profit hospitals (95%CI, −$6.2 to −$4.6 million; *P* < .001); however, for-profit hospitals were not in PHE financial distress even with this lower funding (Table 4).

**Table 5.** Predicted COVID-19 Relief Funding, US Dollars^a^

	Predicted COVID-19 relief funds (95% CI), $	*P* value
Hospital type [reference = short-term acute care]		
Critical access hospital	−3 129 900 (−4 361 974 to −1 897 827)	<.001
Rurality [reference = rural]		
Urban	465 869 (−367 324 to 1 299 061)	.27
Highly urban	4 803 680 (3 615 819 to 5 991 542)	<.001
Health system [reference = no]		
In a health system	−1 191 779 (−2 274 244 to −109 315)	.03
DSH status [reference = no DSH]		
Medicare-only	2 884 907 (1 445 707 to 4 324 107)	<.001
Medicaid-only	1 195 465 (372 872 to 2 018 058)	<.001
Medicare and Medicaid	5 749 664 (4 591 902 to 6 907 427)	<.001
Uncompensated care as a proportion of operating expenses [reference = 0%]		
<5%	−970 380 (−4 035 960 to 2 095 199)	.54
5-<10%	−2 296 752 (−5 370 666 to 777 161)	.14
10-<15%	−4 616 092 (−7 737 303 to −1 494 880)	<.001
≥15%	−4 917 597 (−8 097 087 to −1 738 107)	<.001
Black population [reference = <20%]		
≥20% Black population	1 109 617 (−240 047 to 2 459 281)	.11
Population in poverty [reference = <20%]		
≥20% population below the poverty line	−188 745 (−−1 424 194 to 1 046 704)	.77
Hispanic population [reference = <20%]		
≥20% Hispanic population	862 634 (−282 325 to 2 007 593)	.14
Teaching hospital [reference = no]		
Teaching hospital	8 816 218 (7 507 799 to 10 100 000)	<.001
Ownership [reference = not-for-profit]		
For-profit	−5 409 987 (−6 236 375 to −4 583 599)	<.001
Government	−101 598 (−1 255 696 to 1 052 500)	.86
Weighted net operating income, 2017-2019 [reference = negative]		
Positive	523 442 (−291 051 to 1 337 935)	.21

Abbreviations: DSH, disproportionate share hospital.

^a^ Dollar amounts represent predicted COVID-19 relief funds relative to the reference category, not the predicted absolute amount of COVID-19 relief.

Conversely, the following hospitals received more COVID-19 funding relative to their counterparts. Highly urban hospitals received on average $4.8 million more in COVID-19 relief funding compared with rural hospitals (95% CI, $3.6 million-$6.0 million; *P* < .001). Hospitals with any DSH status received more COVID-19 relief funding than those with no DSH status. Teaching hospitals received on average $8.8 million more in COVID-19 relief funding compared with nonteaching hospitals (95% CI, $7.5 million-$10.1 million; *P* < .001). Pre-2020 negative net operating income was not a significant predictor of receipt of any COVID-19 funding and/or the amount of COVID-19 funding received.

## Discussion

This longitudinal cohort study of US hospitals empirically describes how the COVID-19 pandemic was associated with the finances of acute care and critical access hospitals nationally and evaluates differences by hospital and community characteristics. We find most hospitals did not fare poorly during the first 2 years of the COVID-19 PHE, with 74.8% having a positive operating income across 2020/2021, and only 16.3% characterized as having new PHE distress. Indeed, most hospitals improved their financial performance compared with pre-2020. The median within-hospital change in net operating income from pre-2020 to 2020/2021 was $1.9 million and a median 2020/2021 weighted operating margin of 6.5%. To place this in context, MedPAC reports that pre–COVID-19, the all-time high all-payer hospital operating margins averaged 6.4%.^13^

A small minority of hospitals (6.4%) fared substantially worse in 2020/2021 compared with their prior performance and had new negative net operating income; these hospitals suffered considerable financial losses with a median within-hospital decline in net operating income of $8.9 million. Interestingly, a larger minority (17.8%) fared substantially better during 2020/2021 than pre-2020, moving from a negative to a positive net operating income and a median within-hospital improvement of $4.1 million in net operating income.

For 30% of hospitals, COVID-19 funding proved important in mitigating the negative effects of the pandemic on financial performance; the proportion of hospitals that had new PHE distress moved from 46.3% in the absence of COVID-19 funding to 16.3% with COVID-19 funding. However, COVID-19 relief funds may have also helped a substantial proportion of hospitals improve their pre–COVID-19 financial performance. More than three-quarters of the 53.7% of hospitals that did not experience new PHE distress exclusive of COVID-19 relief funds still received such funds, raising questions about the appropriateness of relief funding for these hospitals and how relief funding may be better targeted in the event of a future pandemic. Particularly noteworthy, COVID-19 relief funds may have helped approximately 12% of hospitals dramatically improve their financial performance, as evidenced by the 5.9% of hospitals that moved from negative net operating income pre-2020 to positive net operating income absent COVID-19 relief funds, yet the 17.8% of hospitals that moved from negative net operating income pre-2020 to positive net operating income when including COVID-19 relief funds.

In analyses aimed at understanding predictors of PHE financial distress, we found that hospital location in areas with higher proportions of Hispanic residents was the only independent variable associated with PHE financial distress after receipt of PHE funding. This suggests that COVID-19 relief funding was not sufficient for these hospitals. Other work has also found that hospitals serving underresourced populations were at higher risk of poor financial performance during the pandemic.^14^ However, the present study’s results indicate that COVID-19 relief funds averted financial distress for the following types of hospitals: government-owned hospitals, teaching hospitals, Medicare/Medicaid DSH hospitals, and those serving larger proportions of Black populations.

Conversely, we found without relief funding, for-profit hospitals, and health system-affiliated hospitals were not in financial distress, suggesting that the provision of COVID-19 funding to these hospitals may not have been necessary.

Given that half of all hospitals had 2020/2021 operating margins that exceeded pre–COVID-19 all-time high values for operating margins suggested that COVID-19 relief funds allowed some hospitals to achieve top financial performance, rather than address financial solvency. For example, before 2020, the median operating margin was 2.9% for the hospitals we studied; in 2020/2021 this value grew to 6.5% when including COVID-19 relief funds, moving hospitals to more robust operating margins than before the pandemic.

The literature on hospital financial performance during the PHE is small, and this study adds to a growing body of evidence indicating that during 2020/2021, most hospitals fared well financially,^15^ with financial performance buttressed partly due to COVID-19 relief funding,^16,17^ albeit with concerning financial performance for hospitals serving vulnerable populations^14^.

### Limitations

The present study’s analyses ended in 2021 because data for 2022 were unavailable at the time of analysis. Though this represents the first 2 years of the COVID-19 PHE, results may not generalize after 2021. For example, the HRSA COVID-19 Uninsured Program, which reimbursed health care entities caring for uninsured individuals with COVID-19, was insolvent by fall 2022; this analysis may therefore underestimate the 2022 and 2023 risk of PHE financial distress for hospitals that disproportionately care for larger numbers of uninsured patients.^1^

## Conclusions

Hospitals provide an important medical safety net and represent 31% of all health care spending in the US^18^; for both reasons, it is important to examine how they fared financially during the PHE and the extent to which COVID-19 assistance was well-targeted. In this longitudinal cohort study of US hospitals, although the large majority of hospitals fared well during the first 2 years of the pandemic, partly because of the receipt of COVID-19 relief funds, some hospitals experienced substantially worsened financial health and should continue to be monitored to prevent access problems. From a policy standpoint, COVID-19 funds were especially important to the financial health of hospitals that serve historically underserved, vulnerable patient populations, namely government-owned hospitals, Medicare/Medicaid DSH hospitals, and hospitals serving larger proportions of Black populations. However, this was counterbalanced by the present study’s findings demonstrating that COVID-19 relief funds went to some hospitals that did not need financial support or the amount of funding allocated, consistent with findings from a prior study.^19^ This resulted in moving many hospitals to peak historical operating margins (ie, profitability), rather than simply restoring them to prepandemic operating margins.^3^ It should be underscored that policy makers were required to act quickly to direct COVID-19 PHE funding; however, based on the COVID-19 lessons, it will be important to consider alternative ways of allocating scarce public dollars to support our nation’s health system in crisis. To that end, policy makers should ensure they have the necessary data to estimate the effects and to proactively build models to simulate relief payments and their effects on hospital finances, which could be used to better inform decision-making regarding the allocation of emergency aid.

## Notes

Supplement 1. **eTable 1.** Correlation coefficients for covariates used in regression models

Supplement 2.
Data Sharing Statement
