# Supplementary material for: Changes in US Hospital Financial Performance During the COVID-19 Public Health Emergency
Source: JAMA Health Forum. 2023 Jul 14;4(7):e231928. doi: 10.1001/jamahealthforum.2023.1928 (PMC10349333; doi:10.1001/jamahealthforum.2023.1928)
Supplement: Supplement 1. — eTable 1. Correlation coefficients for covariates used in regression models [file jamahealthforum-e231928-s001.pdf]

Supplemental Online Content

Gidwani R, Damberg CL. Changes in US hospital financial performance during the COVID-19 public health emergency. *JAMA Health Forum.* 2023;4(7):e231928. doi:10.1001/jamahealthforum.2023.1928

eTable 1. Correlation coefficients for covariates used in regression models

This supplemental material has been provided by the authors to give readers additional information about their work.

© 2023 Gidwani R et al. *JAMA Health Forum.*

Appendix Table A1. Correlation Coefficients for Covariates Used in Regression Models

	Hospital Type	Urbanicity	Teaching Hospital	Belongs to a Healthcare System	DSH Status	Uncompensated Care as a Proportion of Operating Expenses	PHE Funding as a Proportion of Operating Expenses	20% or more Black Population	20% or more of Population Below Poverty Line	20% or more Hispanic Population	Ownership Status
Hospital type	1										
Urbanicity	-0.49	1									
Teaching hospital	-0.38	0.39	1								
Belongs to a healthcare system	-0.34	0.29	0.30	1							
DSH status	-0.500	0.18	0.32	0.29	1						
Uncompensated care as a proportion of operating expenses	-0.07	-0.04	-0.07	0.06	0.16	1					
PHE funding as a proportion of operating expenses	0.21	-0.22	-0.15	-0.24	-0.07	-0.01	1				
20% or more Black population	-0.15	0.10	0.11	-0.01	0.11	0.08	0.02	1			
20% or more of population below poverty line	-0.02	-0.13	-0.02	-0.14	0.09	0.13	0.09	0.44	1		
20% or more Hispanic population	0.14	0.24	0.09	-0.05	0.07	0.15	-0.06	-0.04	0.08	1	
Ownership Status	0.18	-0.15	-0.14	-0.36	-0.11	0.10	0.10	0.11	0.14	0.11	1

© 2023 Gidwani R et al. *JAMA Health Forum.*
