# Supplementary material for: Changes in US Hospital Financial Performance During the COVID-19 Public Health Emergency
Source: JAMA Health Forum. 2023 Jul 14;4(7):e231928. doi: 10.1001/jamahealthforum.2023.1928 (PMC10349333; doi:10.1001/jamahealthforum.2023.1928)
Supplement: Supplement 2. — Data Sharing Statement [file jamahealthforum-e231928-s002.pdf]

Data Sharing Statement

Gidwani. Changes in US Hospital Financial Performance During the COVID-19 Public Health Emergency. *JAMA Health Forum*. Published July 14, 2023.
doi:10.1001/jamahealthforum.2023.1928

Data
Data available: Yes
Data types: Other (please specify)
Additional Information: All data are available from the RAND Hospital data team, pursuant to completion of appropriate data use agreements.
How to access data: https://www.hospitaldatasets.org/
When available: With publication

Supporting Documents
Document types: None

Additional Information
Who can access the data: Researchers who proposed use of the data has been approved.
Types of analyses: For any purpose.
Mechanisms of data availability: With a signed data use agreement.
